# A Hydrologic Landscapes Perspective on Groundwater Connectivity of Depressional Wetlands

**DOI:** 10.3390/w12010050

**Published:** 2019-12-21

**Authors:** Brian P. Neff, Donald O. Rosenberry, Scott G. Leibowitz, Dave M. Mushet, Heather E. Golden, Mark C. Rains, J. Renée Brooks, Charles R. Lane

**Affiliations:** 1 Former post-doctoral Research Hydrologist, National Research Program, U.S. Geological Survey, Lakewood, CO 80225, USA; 2 Earth System Processes Division, Water Mission Area, U.S. Geological Survey, Lakewood, CO 80225, USA; 3 Pacific Ecological Systems Division, Center for Public Health and Environmental Assessment, U.S. Environmental Protection Agency, Corvallis, OR 97333, USA; 4 Northern Prairie Wildlife Research Center, U.S. Geological Survey, Jamestown, ND 58401-7317, USA; 5 Center for Environmental Measurement and Modeling, Office of Research and Development, U.S. Environmental Protection Agency, Cincinnati, OH 45268, USA; 6 School of Geosciences, University of South Florida, Tampa, FL 33620, USA

**Keywords:** hydrologic landscapes, landscape hydrology, depressional wetlands, geographically isolated wetlands, hydrologic connectivity, wetland connectivity, indirect groundwater connectivity, direct groundwater connectivity, VS2DI, groundwater-surface water interactions

## Abstract

Research into processes governing the hydrologic connectivity of depressional wetlands has advanced rapidly in recent years. Nevertheless, a need persists for broadly applicable, non-site-specific guidance to facilitate further research. Here, we explicitly use the hydrologic landscapes theoretical framework to develop broadly applicable conceptual knowledge of depressional-wetland hydrologic connectivity. We used a numerical model to simulate the groundwater flow through five generic hydrologic landscapes. Next, we inserted depressional wetlands into the generic landscapes and repeated the modeling exercise. The results strongly characterize groundwater connectivity from uplands to lowlands as being predominantly indirect. Groundwater flowed from uplands and most of it was discharged to the surface at a concave-upward break in slope, possibly continuing as surface water to lowlands. Additionally, we found that groundwater connectivity of the depressional wetlands was primarily determined by the slope of the adjacent water table. However, we identified certain arrangements of landforms that caused the water table to fall sharply and not follow the surface contour. Finally, we synthesize our findings and provide guidance to practitioners and resource managers regarding the management significance of indirect groundwater discharge and the effect of depressional wetland groundwater connectivity on pond permanence and connectivity.

## 1. Introduction

As their name implies, depressional wetlands are wetlands that form within depressions on the landscape where they have limited surface-water connectivity and are more dependent on atmospheric exchanges than other wetland types [1,2]. Interest in the hydrologic connectivity of depressional wetlands has accelerated in recent years, largely in response to the need for scientific information to guide wetland management.

A 2003 special issue of *Wetlands* provided a historical orientation to the legal and scientific context of depressional wetlands with limited surface-water connections [3], including a synthesis of the then-current scientific understanding and a guide to future research [4]. In the special issue, these wetlands were described as “geographically isolated.” However, recent research has shown that even the surface-water connectivity of so-called “geographically isolated” wetlands has been underestimated [5,6], and the term has recently been described as being a misnomer [7]. A little over a decade after the special issue of *Wetlands*, the U.S. Environmental Protection Agency published a review and synthesis of over 1200 peer-reviewed scientific publications on the connectivity of headwater streams and wetlands [8]. This report identified connectivity of non-floodplain, primarily depressional, wetlands as a key gap in scientific understanding, particularly with regard to how connectivity varies over space and time. Lane, et al. [9] provided an updated review of the literature on non-floodplain wetlands.

Other studies are noteworthy for providing insights to the variable hydrologic regimes of depressional wetlands [10,11]. Intermittent connectivity to downstream waterbodies can be through surface water by ‘fill and spill’ [12–14] or ‘fill and merge’ [5] events. Groundwater can also flow between depressional wetlands and to downgradient waterbodies [15,16]. Hayashi, et al. [17] applied a water-balance perspective to simultaneously consider surface-water and groundwater connectivity and explain wetland permanence.

With regard to the effects of wetlands on other waterbodies, McLaughlin, et al. [18] modeled how depressional wetlands in Florida hydraulically moderate baseflow in downgradient waterbodies, even though no water passes from the wetland to the downstream waterbody. Golden, et al. [19] used a hybrid statistical and process-based modeling approach to quantify the cumulative effect of depressional wetlands on downgradient streamflow. Lane, et al. [9] provided a review of the hydrologic, physical, and chemical effects of non-floodplain wetland connectivity on downgradient aquatic systems. Rains, et al. [11] proposed that wide variability in wetland connectivity cumulatively creates a specific effect on the hydrologic functioning of downstream waterbodies.

The challenge remains to develop broad, perhaps universally applicable, insights into the hydrologic connectivity of depressional wetlands. Some studies have attempted to link landscape form, geology, and climate to conceptualize wetland persistence and sensitivity to land use [20,21]. Leibowitz, et al. [22] were notable for explicitly applying the hydrologic landscapes theoretical framework [23] to conceptualize the factors affecting hydrologic connectivity across a wide range of landscapes. The hydrologic landscapes theoretical framework was developed by Tom Winter in the 1980s and 1990s to provide a relatively simple framework to explain the flow of water across any given landscape, from highlands to lowlands, through groundwater or surface water [24–26]. Winter published two widely cited papers illustrating the hydrologic landscapes concept by explaining groundwater flow through six conceptual, two-dimensional landscapes [23,27]. Leibowitz, et al. [22] used these same landscapes to illustrate their framework for understanding connectivity. This approach is elegant in its understandability and use of a universally applicable theoretical framework with potential to conceptualize all aspects of hydrologic flow and connectivity in a landscape, including the interplay between surface water and groundwater.

While innovative and useful, the Leibowitz, et al. [22] study left important opportunities for future research. First, their study used the hydrologic landscapes framework to explain the relative magnitude of connectivity between floodplains and floodplain wetlands with rivers in diverse landscapes. They did not consider the connectivity of depressional wetlands existing within a landscape. Second, the focus of the Leibowitz study was the connectivity between headwaters and wetlands with rivers *downstream*. An opportunity exists to consider the importance of *upgradient* connectivity. Third, the Leibowitz paper effectively used the hydrologic landscapes framework to help explain diverse connectivity patterns reported in the literature but did not try to advance the hydrologic landscapes theoretical framework. This serves as the point of departure for the present study.

Our purpose in this study is to use the hydrologic landscapes perspective to develop insights into the hydrologic connectivity of depressional wetlands that are broadly, or universally, applicable. We define hydrologic connectivity to be the flow of water through various features of a landscape such as uplands, lowlands, wetlands and other surface water bodies and the ground. Our focus is primarily on groundwater flow and how groundwater interacts with surface waters. We numerically simulate hydrologic connectivity within five of the six generic landforms presented in Winter [23]: playa, plateau, mountain valley, riverine valley, and coastal terrain. We then insert depressional wetlands into these generic landforms and repeat the simulation exercise. We did not reproduce the sixth landform in Winter [23], hummocky terrain, largely because this landform is comprised of depressional wetlands. We do, however, return to the hummocky landform in our discussion and show how the lessons learned in our modeling apply to the patterns of groundwater flow in the hummocky landform in Winter [23].

In our numerical simulations, we evaluate the linkages between groundwater and surface water flows within the hydrologic-landscapes framework, then we evaluate how the presence of depressional wetlands affects hydrologic connectivity. Our evaluation has both qualitative and quantitative character. We assess the patterns and changes in flowpaths in the landscape both visually and by quantifying groundwater discharge to the surface and to depressional wetlands in the landscape.

### 1.1. The Hydrologic Landscapes Theoretical Framework

**Figure 1.**
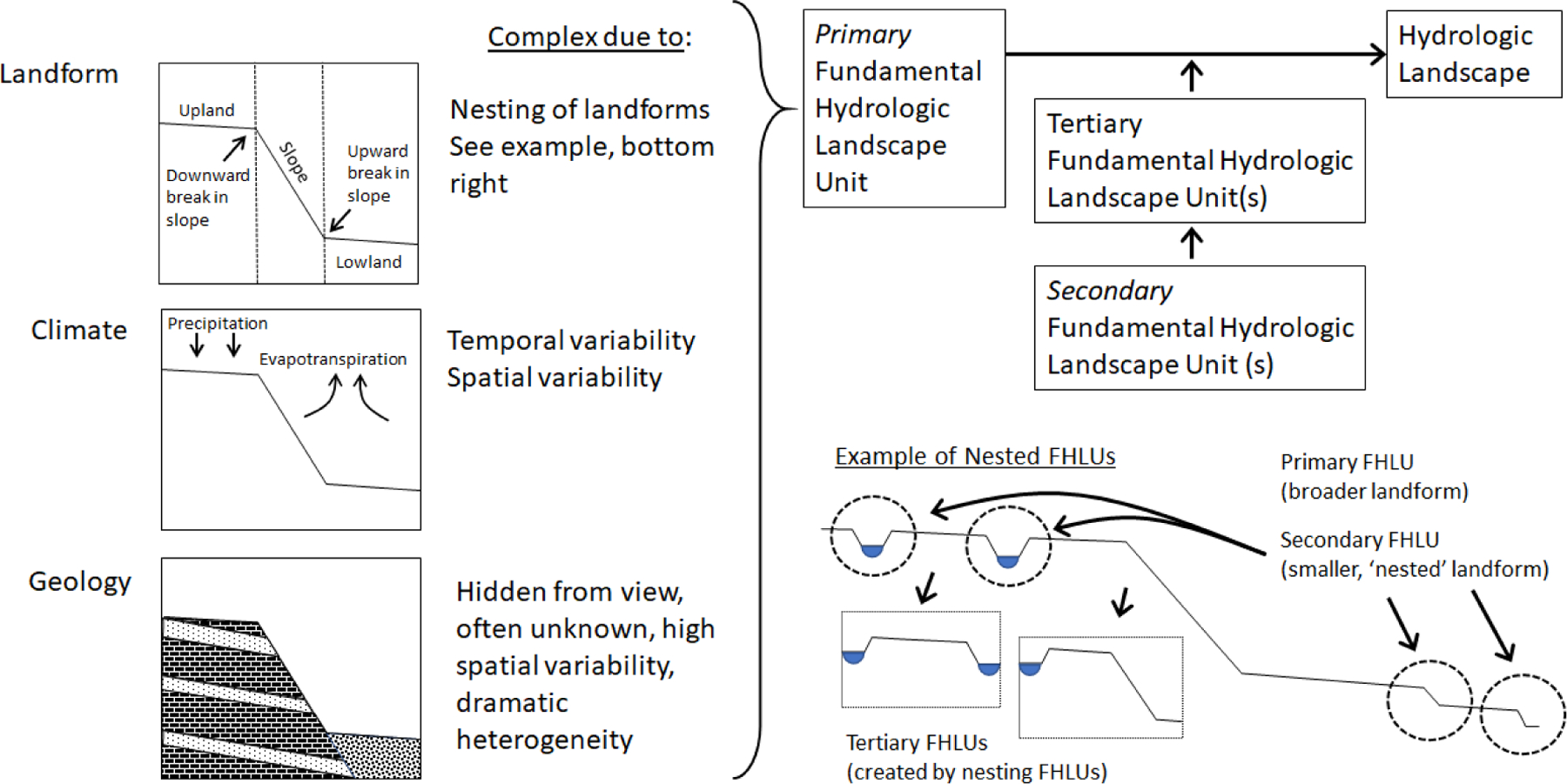
Conceptualization of a hydrologic landscape, consisting of one or more fundamental hydrologic landscape units (FHLUs). Each FHLU consists of a landform, climate, and geology. The right side of the figure depicts how FHLUs can become nested within a hydrologic landscape.

The hydrologic landscape theoretical framework describes the flow of water through *fundamental hydrologic landscape units* (FHLUs). Each FHLU has three components, a landform, climate, and geology [23,27]. The *landform* is the physical shape of the land and consists of an upland and a lowland connected by a slope. *Climate* is represented as the combined effect of precipitation and evapotranspiration (ET). *Geology* controls how water flows across or through the landscape in response to climate. When considered together, landform, climate, and geology comprise an FHLU (Figure 1).

In practice, FHLUs exist at many scales. For example, a landform, defined as an upland and lowland divided by a slope, could be considered as the top of a plateau, an adjacent river, and the valley slope dividing the two. When combined with geology and climate this could be considered a plateau FHLU. However, a landform could also be considered as being the upland area immediately above a depressional wetland, the slope of the depression, and wetland itself serving as the lowland. When this landform is combined with climate and geology it could be correctly considered a depressional wetland FHLU. Infinite possibilities of FHLUs exist. To further complicate matters, FHLUs are often embedded within one another. In the above examples, a depressional wetland FHLU could exist within a plateau FHLU. This exact scenario is considered in Figure 1 and later in our simulations.

The embedding of one FHLU within a relatively larger FHLU is described as *nesting* [23,27]. In the hydrologic landscape context, the nesting concept is unrelated to the concept of nested streamflow gauging stations or sampling sites and simply refers to one or more smaller FHLUs occurring within a larger FHLU. The configuration of a *hydrologic landscape* is defined as either a variant of a FHLU or as the sum of all nested FHLUs. For example, Winter [27] describes riverine hydrologic landscapes as having a landform “characterized by relatively broad lowlands that have smaller fundamental landscape units such as terraces nested within them.” We expand here on the sparse hydrologic landscapes nomenclature to define the *primary FHLU* as a spatially larger unit, into which one or more *secondary FHLUs* are embedded, or nested. Depressional wetlands and terraces are common types of secondary FHLUs. The nesting of FHLUs often causes other, less obvious, landscape features with a highland and lowland divided by a slope, which we term *tertiary FHLUs* to draw attention to the nature of these features as being a byproduct of FHLU nesting. Tertiary FHLUs exist between or adjacent to secondary FHLUs such as depressional wetlands (Figure 1). A hydrologic landscape is the sum of all nested FHLUs.

In Figure 1, the broader, primary FHLU landform is depicted with a clear upland and lowland, divided by a slope. Four secondary FHLUs exist within the broader primary FHLU—two depressional wetlands and two terraces. The depressional wetland secondary FHLUs result in two areas that have their own highland and lowland, divided by a slope, shown in the two box insets labeled as tertiary FHLUs in Figure 1. In the leftmost tertiary FHLU, the two wetlands form the upland and lowland, and the slope actually rises and then falls in elevation between the two wetlands. In the rightmost tertiary FHLU in Figure 1, the wetland serves as the upland, the primary FHLU lowland serves as the lowland, and the slope again rises and falls between the two. When visually inspecting a landscape, it is convenient to first define the broad landform as the primary FHLU; second, to identify the embedded features such as depressional wetlands or terraces and define these as secondary FHLUs; and third, to focus on the less obvious tertiary landforms that are caused by the existence of secondary FHLUs.

## 2. Materials and Methods

The water table is crucial for determining hydrologic connectivity of wetlands. As a general rule, groundwater will not flow from a depressional wetland when surrounded by a mounded water table unless special circumstances exist [16,28,29]. The current study combined relatively simple geology and climate conditions to simulate groundwater flow and water table contour for five of the hydrologic landscapes presented in Winter [23]—playa, plateau, mountain valley, riverine valley, and coastal terrain. We chose these five landscapes to illustrate the hydrologic connectivity of depressional wetlands in a range of settings found throughout North America. In our simulations, we held the climate and geology variables as constant as possible while varying the landform to simplify our analysis of the effects of landform on connectivity of depressional wetlands. Later in the discussion section, we return to the consequence of variability in geology and climate.

**Figure 2.**
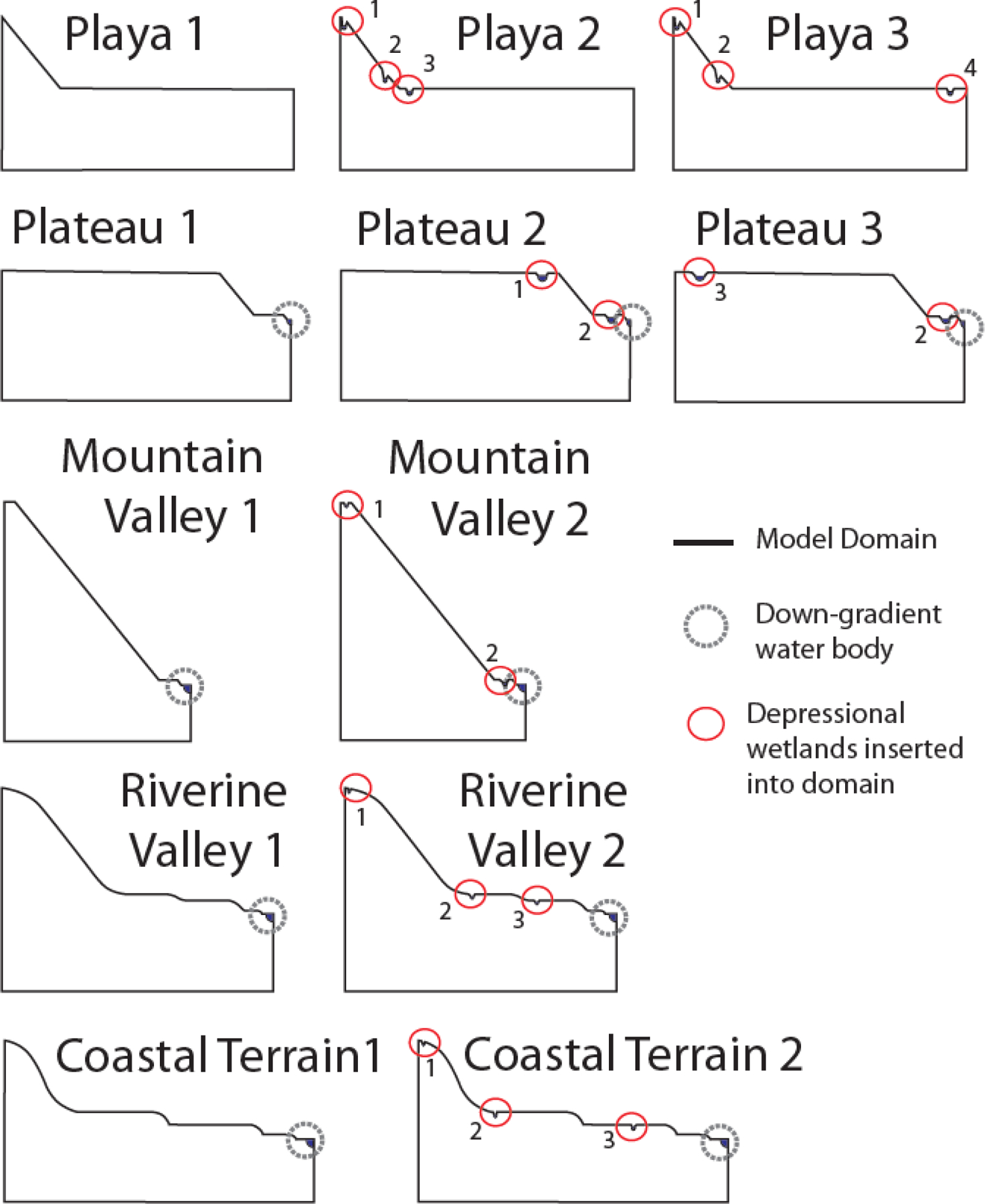
Model domains in this study. Depressional wetlands are numbered.

Groundwater flow was simulated using the VS2DI model, described below, through each of the five domains presented in Winter [23,27]. Additional simulations were run using a second set of domains created by inserting depressional wetlands into various parts of each domain (Figure 2). There is an infinite combination of potential wetland locations and number. The location and number of depressional wetlands in the second set of domains were chosen to convey how a range of wetland locations affects the flow of water through each domain. Groundwater flow and connectivity were simulated in each domain.

Tom Winter provided few details of his conceptual, generic landscapes. No specifics were provided regarding the dimensions of the domains, climatic conditions, or the geologic makeup of the domains other than being homogenous. Additionally, no detail was provided on how, or even if, the flow through each conceptual domain was numerically simulated. As a result, we used reasonable estimates of these variables in our simulations, and trial and error, to simulate a water table similar to that depicted in Winter [23,27]. With regard to the precise height and length dimensions of each model, we sought to create ‘typical’ dimensions for each landform relative to one another as depicted in Winter’s work [23,27]. For example, mountains are typically taller and less expansive in the horizontal dimension than coastal landforms, and our chosen model dimensions reflect that. We chose moderately conductive, homogenous and isotropic geology and a net recharge (precipitation less evaporation) to represent a climatic condition within the range of what is commonly reported in North America. If our simulations reproduced a water table reasonably close to that reported in Winter [23,27], we stopped there.

The dimensions, climatic conditions, and geologic makeup of our model domains are provided in Table 1. The other decisions made regarding the model setup are provided in the following paragraphs. All models and simulation results presented here are archived and available for download at https://doi.org/10.23719/1504529. A supplemental to this article also contains our models, results, and instructions to rerun the simulations and reproduce our results. Our purpose in this study was to draw broad lessons by conceptually modeling a range of hydrologic landscapes with and without embedded depressional wetlands, not to perfectly mimic the flows depicted in Winter [23,27]. In this context, we used the Winter [23,27] studies as a guide.

All simulations were performed using the Variably Saturated 2-Dimensional numerical model (VS2DI). VS2DI is a fully distributed model and uses a finite difference approach to solve the Richards equation for two-dimensional flow through unsaturated and saturated sediments [30,31]:

(1) $$\frac{\partial \left(\theta \left(h\right)+sS_{s}H\right)}{\partial t}=\nabla \cdot K\left(h,T\right)\nabla H+q,$$

where *θ*(ℎ) is volumetric soil water content, *h* is pressure head, *s* is saturation, *S*_*s*_ is specific storage, *H* is total head (*H* = *h*-*z*, with *z* being the positive downward vertical coordinate), *K*(*h*,*T*) is the hydraulic conductivity tensor (assumed to be aligned with the coordinate axes and a function of temperature when heat transport is simulated), *T* is temperature, and *q* is the source/sink function. Healy and Essaid [30] also provided explanations of an alternate expression of the hydraulic conductivity tensor and how this equation has been modified from the original Richards equation to enable the simulation of the flow in both unsaturated and saturated zones.

The VS2DI model was selected for its ability to simulate flow in the unsaturated and saturated zones and allow the water table to adjust to fluxes in the model parameters. In addition, this program provides the ability to visually compare results between domains using a graphical user interface (GUI). Finally, the relative speed and ease of use of VS2DI allowed us to simulate many more scenarios than would have been possible with other programs.

For the geologic substrate, we assigned parameters using generic geologic types. In Table 1, the parameters for soil classes followed by “CP” were documented in Carsel and Parrish [32], and all others were described in Lappala, et al. [33]. We used silty clay CP for the geologic parameters for all playa simulations and silt-loam in simulations of other domains. The parameters for these geologic classes are available in the VS2DI model software and we encourage curious readers to download, inspect, and run our models for themselves.

The model grid cell sizes were selected by starting with relatively coarse cells and testing finer sizes until further reduction did not alter the groundwater flow patterns observed in the simulation results [34]. Ultimately, cell size is a tradeoff between model fidelity and computational demand, where model fidelity is the degree to which the actual attributes and processes of the system are accurately represented [35]. The cell sizes used in this study (Table 1) were deemed to be an appropriate compromise and sufficient for the study purpose of developing broadly applicable insight to wetland hydrologic connectivity.

The boundary conditions for all the model domains were assigned to produce results approximately consistent with Winter [23]. No-flow boundaries were assigned on the bottom and sides of each domain. Potential seepage faces [36] were assigned as needed to promote model stability at upward breaks in slope, adjacent to water bodies. In the case of the Plateau landscapes, promoting model stability while maintaining water table and flow patterns similar to those in Winter [23] required assigning a potential seepage face from the downward break in slope extending to the downgradient waterbody. Our use of seepage face boundary conditions near upward breaks in slope did not constrain seepage to these locations, as our model does permit seepage through specified flow (recharge) boundaries. However, use of the seepage face boundary conditions promoted model stability, and more realistically depicts seepage faces that are commonly observed at upward breaks in slope. The remaining portions of the top boundary were assigned a specified flux into the domain in a vertical direction, which is intended to represent net groundwater recharge (infiltration–evapotranspiration). The one exception to this is that the lower portions of the Playa domains were assigned a specified pressure head equal to zero to reproduce a thin unsaturated zone near the surface and to promote model stability.

The boundary condition for waterbodies was handled two ways. First, a specified total head boundary was assigned to low-lying water bodies (typically the modeled drain) and depressional wetlands extending downward to or below the depression-free FHLU water table. The specified total head was assigned to be equivalent to the surface elevation of ponded water in a depression. However, we found the specified head boundary condition to be unrealistic for ponded water in depressions existing above the normal water table. This condition caused an unrealistic water table to form, saturating up to tens of meters of the unsaturated zone with great and unsustainable (i.e., the wetland would soon become dry) amounts of downward seepage from the wetland. To produce more plausible results, we assigned a specified recharge boundary to these depressional wetland areas equal to one order of magnitude greater than that of the surrounding upland area.

Our decision to use a specified head boundary for low-lying water bodies necessitated the assumption that water seeping upward to the wetland is eliminated through ET, surface water outflow, possibly ‘spill’ surface water outflow, or groundwater outflow in the case of a flow-through wetland. All four of these conditions are features of real landscapes we regularly observe. This is not readily apparent in our 2D model figures and we acknowledge this here.

Our choice of a specified head boundary condition for low-lying wetlands caused the bending of groundwater flow from local and regional flow paths toward the wetland. Similar bending of groundwater flow toward depressional wetlands has been reported in field studies. For example, consider the water table elevation shown in Rains, et al. [37, see Figure 4]. This figure, and study, shows a water table which is warped towards the wetland complex at a surface-water outlet, where outflowing surface water depresses the wetland stage and therefore the local water table. The water table in this case resembles a cone of depression, but with an “outlet” on the downgradient side. This situation, in our experience, is typical in landscapes with any significant relief. The topographic slope in Rains, et al. [37] is approximately 0.02, the average slopes in our domains range from 0.015 to 0.105.

We considered an alternative boundary for low-lying wetlands—a seepage face boundary, where the model determines where the water table intersects the depression, a point at which we could declare the wetland pond elevation. A condition where the seepage-face boundary is likely better is where relief is very low, and the goal was to show seasonal oscillation between wetland recharge and discharge conditions driven by variability in runoff/precipitation and ET. We often see these switches between recharge–discharge conditions, with recharge–discharge neutrality at the crossovers and perhaps overall. Table 2 in Nilsson, et al. [38] described the case where wetlands are recharging local groundwater during the wet season and are receiving local groundwater discharge during the dry season. The landscape slope in the Nilsson case was close to zero. It would be difficult or impossible to model this oscillating condition and perhaps the best way to simulate it would be to simply let the wetland stage be set by the water table.

Ultimately, both types of head boundaries for low-lying depressional wetlands have value and validity, provided they are interpreted correctly. Our purpose was to develop broadly applicable insight into the connectivity of depressional wetlands and we judged the constant-head boundary approach to better simulate the type of depressional wetland behavior we see in the field.

Simulations were run to steady state using homogenous isotropic geology. By maintaining uniform, relatively simple geology and climate conditions in our simulations, we intentionally isolated the effect of the landform on groundwater flow patterns. This approach is similar to that used in Winter [23,27], who depicted homogenous geology and steady-state conditions in developing the generalized hydrologic landscape figures. Furthermore, we used the same geologic and climatic conditions for all of the hydrologic landscapes, with the exception of the Playa landscapes. Geology and net recharge in the Playa landscapes were modified modestly to permit model stability while replicating the results in Winter [23,27] as described below.

For model domains without depressional wetlands, simulations were conducted using geologic substrate and net-recharge conditions suitable to replicate the water table and groundwater flow depicted in the generalized hydrologic landscape figures in Winter [23,27] as closely as possible. Depressional wetlands were inserted into the second set of model domains (Figure 2) and simulations were run using the same geology and climatic conditions. Readers can download and inspect our groundwater-flow models from a supplemental to this article or by accessing a separate publicly available archive at https://doi.org/10.23719/1504529.

## 3. Results

The results of our groundwater flow simulations are generally close to the water table contours and groundwater flow depicted in the iconic hydrologic landscapes in Winter [23]. Notable exceptions to this statement exist, such as the thin unsaturated zone reported in most of the Winter [23,27] landscapes and are explained in the Discussion section. The water table in our simulations closely matched those reported in Winter [23,27]; the water table declined in elevation at downward breaks in slope and rose to the top of the model domain at upward breaks in slope. However, in all simulations we found a large majority (range 48%–100%, average 93.5%) of groundwater discharges to the surface at any particular concave-up break in slope (Table 2). Our use of seepage face boundary conditions near upward breaks in slope did not constrain seepage to these locations, as our model does permit seepage through specified flow (recharge) boundaries.

In all cases, the groundwater connectivity of depressional wetlands inserted into generic hydrologic landscapes depended on the surface elevation of ponded water in a wetland relative to the surrounding water table. However, a key aspect of our results is that in some cases, described below, the addition of one or more depressions to the model domain affects the elevation of the water table sufficiently to profoundly affect the groundwater and surface-water connectivity of the added depressional wetland(s).

### 3.1. Playas

**Figure 3.**
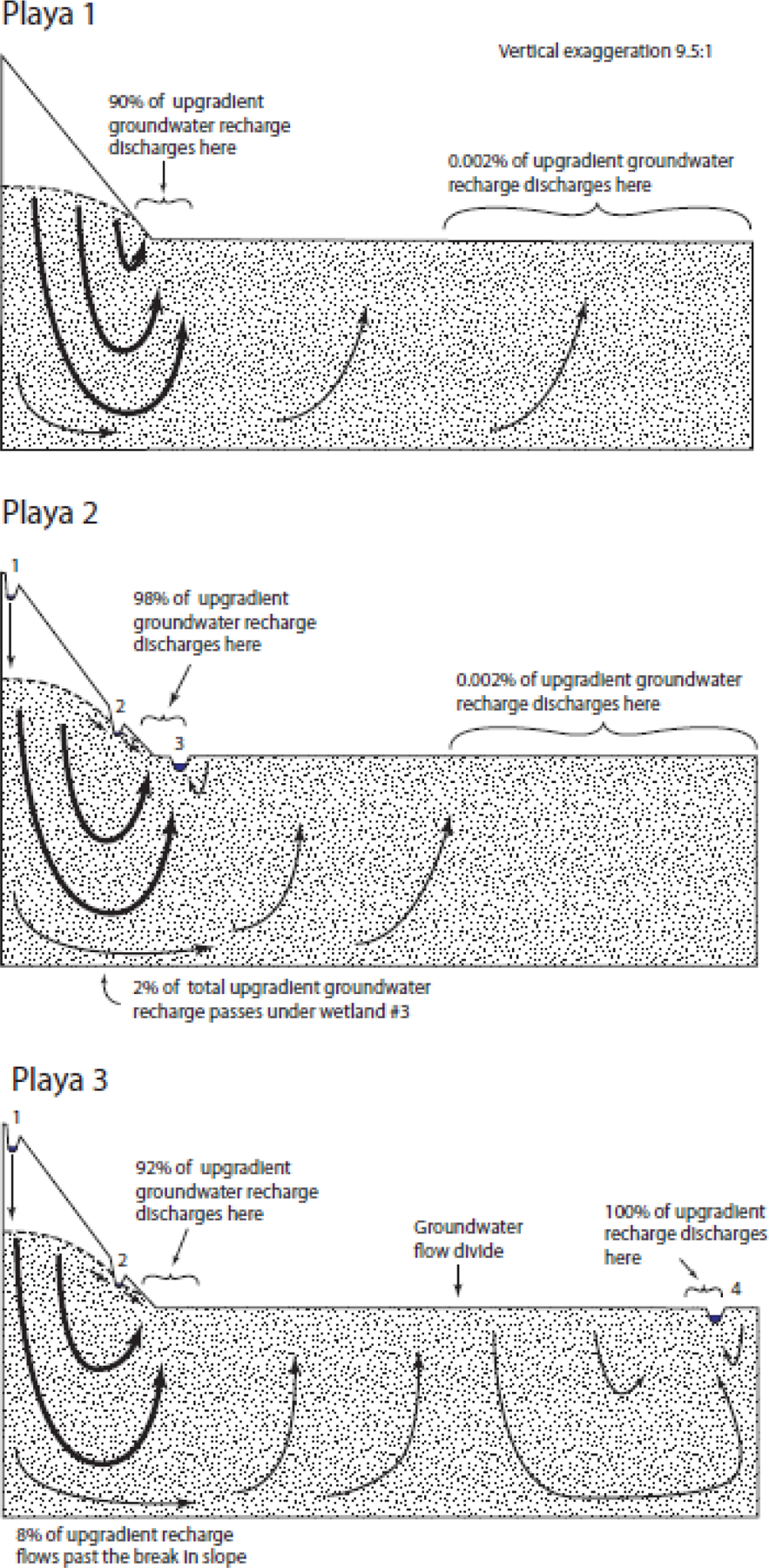
Simulated flow through the Playa landscapes.

The simulated groundwater flow through the generic Playa landscape (Playa 1; Figure 3) showed predictable flow paths from highlands to lowlands. However, 90% of recharge from upland areas discharged within 500 m of the upward break in slope. Discharge decreased rapidly as distance from the break in slope increased (Table 2). In fact, only 0.002% of all groundwater discharge occurred on the more distant half of the lowland. It is important to recognize that groundwater discharge at the upward break in slope either evaporates or continues flowing downgradient as surface water. As discussed further in the Discussion section, real-world playas are often arid and ET draws the water table well below land surface, causing many depressional wetlands to be recharge wetlands.

The Playa 2 landscape had three depressional wetlands inserted into the Playa 1, generic landscape, Wetland 1, 2, and 3. Wetland 1 was positioned near the top of the playa highland. This wetland was well above the water-table elevation at this location and recharged groundwater, causing the water table to rise somewhat relative to the Playa 1 landscape without wetlands. Wetland 3 was positioned slightly downgradient of the upward break in slope, with a water surface below the surrounding water table. This wetland received groundwater discharge from all sides and did not connect to downgradient waterbodies by groundwater. Wetland 2 is a more complex case. It is situated in a location that had a water table near or at ground level in the generic Playa 1 landscape. Thus, one might expect the water surface of Wetland 2 to be below the surrounding water table and to receive groundwater from all sides. However, the water table on the downgradient side of Wetland 2 does not rise with the land surface. It instead decreases in elevation to form a continuous downward slope away from Wetland 2, causing it to become a flow-through wetland by receiving groundwater discharge on the upgradient side and recharging groundwater on the downgradient side. With regard to surface water connectivity, it is possible that any of the three wetlands could fill and spill and connect to downgradient waterbodies by surface water. Wetland 3 is the most likely of the three wetlands to overfill its depression and spill for two main reasons. First, by virtue of the lower position of Wetland 3 on the landscape relative to Wetlands 1 or 2, Wetland 3 has a larger watershed and therefore is likely to receive more runoff. Second, Wetland 3 is the only wetland receiving groundwater from all sides and is therefore is likely to receive more groundwater inflow.

The Playa 3 landscape is similar to the Playa 2 landscape, except Wetland 3 is moved to a position on the lowland far away from the break in slope and renamed Wetland 4. Wetland 4 exists below the surrounding water table and receives groundwater discharge on all sides. However, the presence of Wetland 4 created a groundwater flow divide near the center of the lowland portion of the Playa 3 landscape (Figure 3). This flow divide completely eliminates the groundwater flow from the playa upland to the far end of the lowland and Wetland 4. However, local groundwater flow to Wetland 4 from the nearby lowland was significant and vastly exceeded the exceptionally small groundwater flow in the far end of the Playa 1 and Playa 2 landscapes.

### 3.2. Plateau

**Figure 4.**
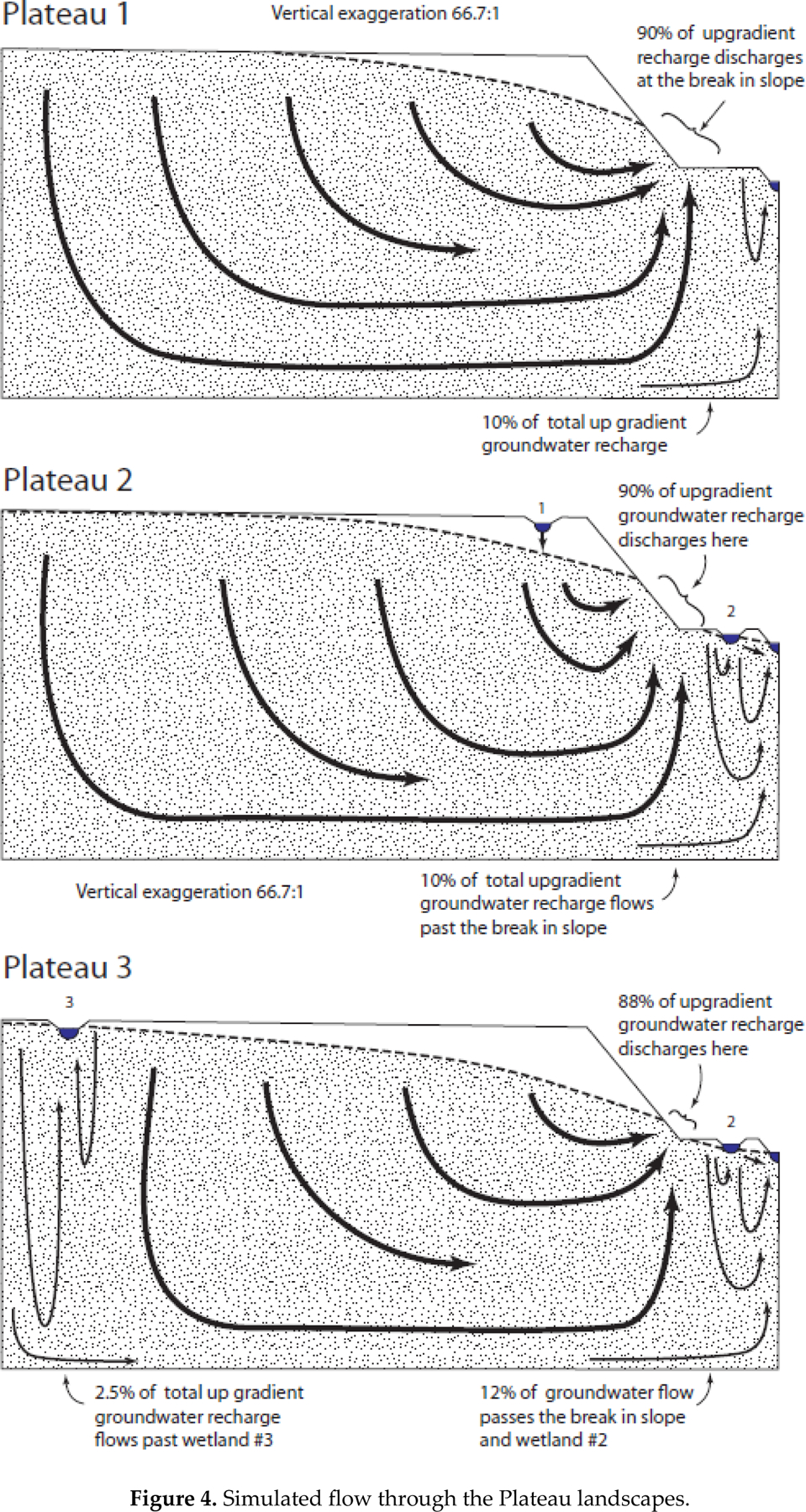
Simulated flow through the Plateau landscapes.

The Plateau landscapes (Figure 4) experienced large groundwater discharge near the upward break in slope. Similar to the Playa landscapes, 88%–90% of the groundwater flow discharged near the upward break in slope and potentially continued toward the downgradient waterbody as surface water (Table 2). The seepage face on the uphill side of the break in slope was much wider in the Plateau 1 and Plateau 2 landscapes relative to the Plateau 3 landscapes. Discharge near the break in slope was roughly proportional to the extent of the seepage faces, as 61%, 63%, and 55% of groundwater discharge occurred uphill of the break in slope, respectively.

Insertion of depressional wetlands into the Plateau 2 landscape influenced groundwater flow much like in the Playa landscapes. In the Plateau 2 landscapes, Wetland 1 exists well above the water table and recharges groundwater. Wetland 2 exists in a groundwater discharge area and is in an area with a relatively high water table in the generic landscape. Nevertheless, the water table on the downgradient side of Wetland 2 did not form a water-table mound but instead sloped continuously downward, indicating flow toward the model drain. This caused Wetland 2 to be a flow-through wetland. In the Plateau 3 landscape, Wetland 1 was moved to the far-left side of the domain and renumbered Wetland 3. The simulated stage of Wetland 3 was lower than the surrounding water table and the wetland, and therefore received groundwater discharge from all sides. With regard to surface-water connectivity, it is possible for any of the wetlands in the Plateau landscapes to fill and spill.

### 3.3. Mountain Valley

**Figure 5.**
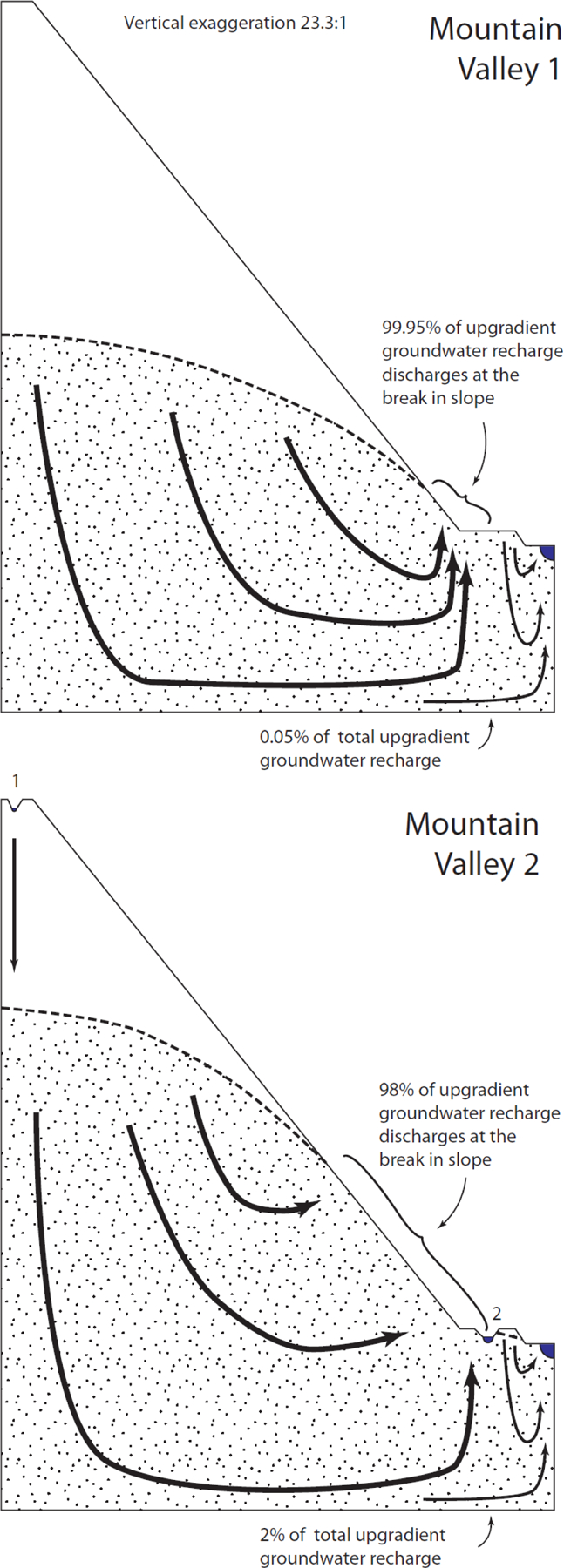
Simulated flow through the Mountain Valley landscapes.

The generic Mountain Valley 1 landscape (Figure 5) was similar to the generic Plateau and Playa landscapes, except nearly all water recharged in the uplands discharged at the upward break in slope, with only 0.05% of upland recharge continuing to the downgradient waterbody as groundwater (Table 2). In the Mountain Valley 2 landscape, Wetland 1 was inserted near the highest position in the landscape. This wetland was many tens of meters above the water table and simply recharged groundwater, causing a slight but largely inconsequential increase in the elevation of the water table. Inserting Wetland 2 at the upward break in slope provided an example of a depressional wetland that one might expect to have a water level below the surrounding water table and receive groundwater discharge from all sides. However, the water table on the downgradient side of Wetland 2 slopes continuously downward toward the model drain, allowing Wetland 2 to be a flow-through wetland. All other patterns of groundwater flow were similar to the generic, wetland-free, Mountain Valley 1 landscape.

### 3.4. Riverine Valley

**Figure 6.**
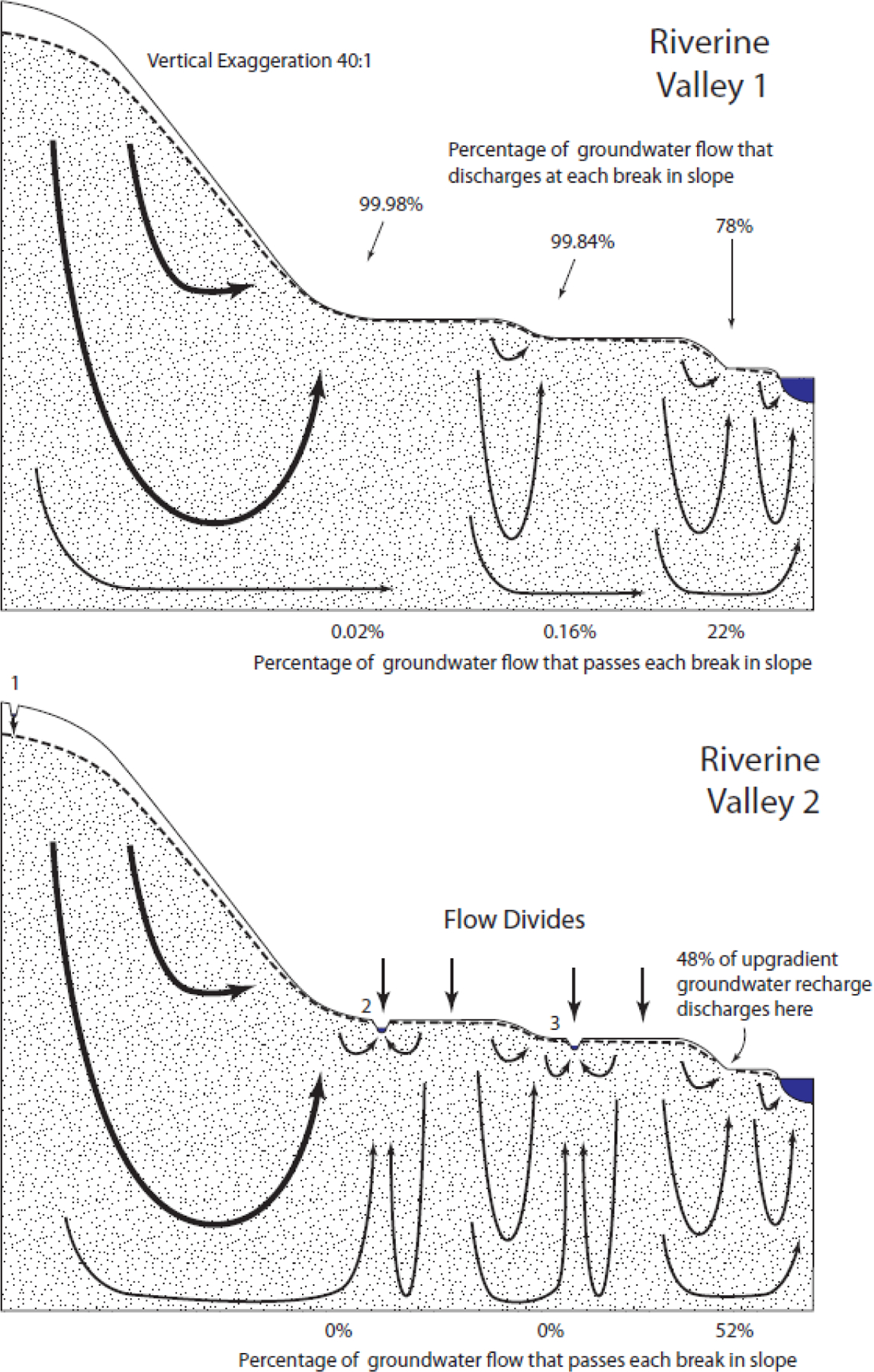
Simulated flow through the Riverine Valley landscapes.

The generic Riverine Valley 1 landscape (Figure 6) had three upward breaks in slope, causing relatively complex groundwater flow patterns. Nearly all of the groundwater discharged at the two highest upward breaks in slope (Table 2). In a technical sense, some small groundwater connection exists between the upland area and the downgradient water body. In a practical sense, the groundwater connection is likely insignificant. As with all other hydrologic landscapes, groundwater discharges to the surface and then either evaporates or continues its journey to downgradient water bodies as surface water. Adding depressional wetlands in the Riverine Valley 2 landscape created several groundwater-flow boundaries and relatively complex flow paths. Groundwater flow from upland areas to downgradient waterbodies is completely eliminated.

### 3.5. Coastal Terrain

**Figure 7.**
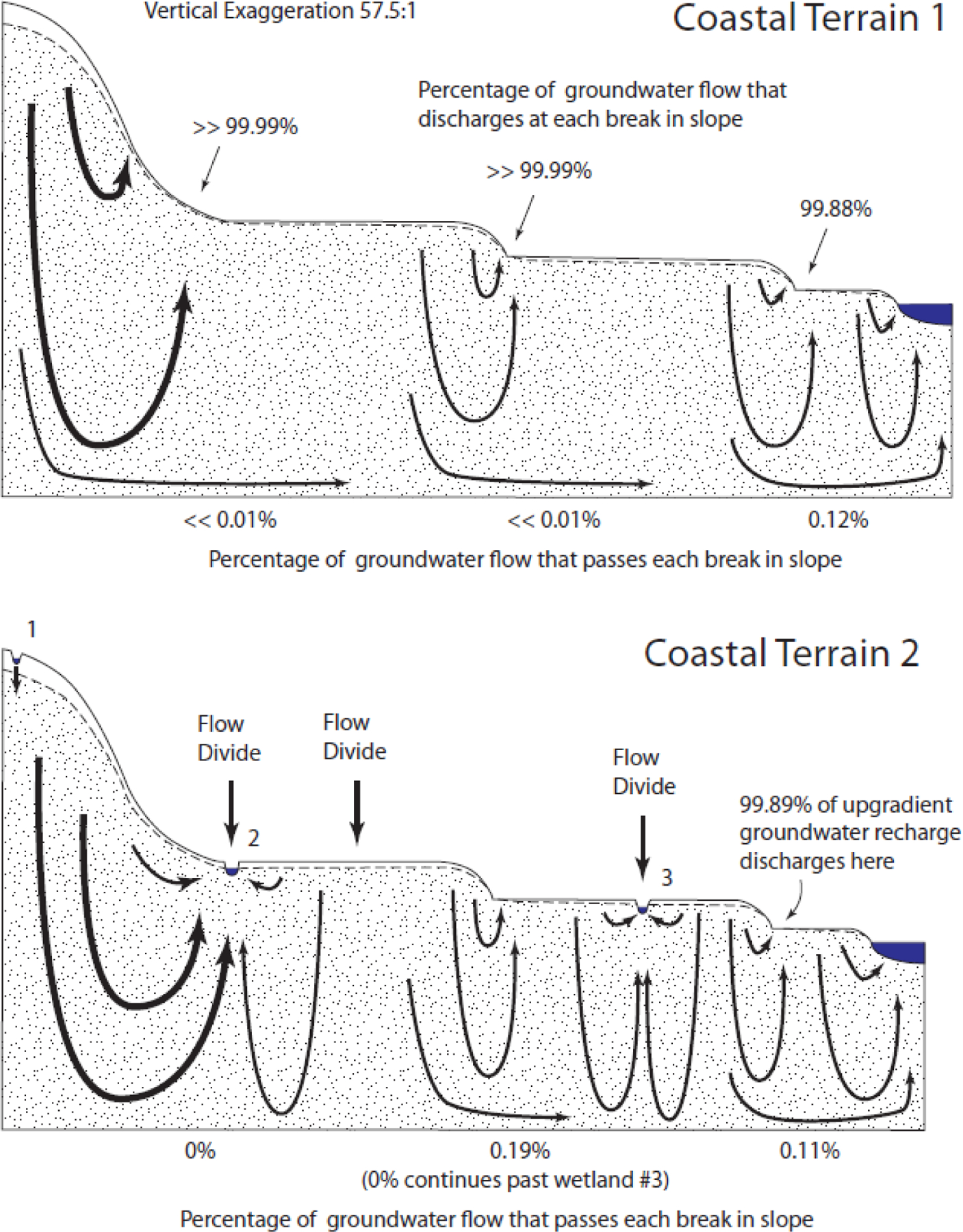
Simulated flow through the Coastal Terrain landscapes.

Groundwater flow patterns in the Coastal Terrain 1 and 2 landscapes (Figure 7) followed nearly the same patterns observed in the Riverine Valley 1 and 2 landscapes, respectively. Notably, the discharge at the upward breaks in slope was very nearly 100% (Table 2).

Readers can reproduce and manipulate the patterns we observed by accessing the publicly available archive of our groundwater-flow models at https://doi.org/10.23719/1504529 or in a supplemental to this article, running the models and then modifying the parameters in additional simulations. Modifying individual depressions to be deeper or shallower than the water table at a location is particularly illustrative.

## 4. Discussion

### 4.1. Groundwater Discharge at the Upward Break in Slope

The point where a downhill slope breaks, or begins to ‘flatten out’, is termed an upward break in slope (Figure 1). The existence of groundwater discharge at the upward break in slope has been widely acknowledged in the literature and is likely intuitive to many hydrologists [23,26,39,40]. However, to our knowledge, the proportion of groundwater flow that discharges at a break in slope has not explicitly been quantified or emphasized. In our simulations, a large majority of groundwater flow, between 48% and very nearly 100%, discharged to the surface at upward breaks in slope in the landscapes modeled in this study. Direct groundwater flow from an upland area in a landscape to a downgradient waterbody or drain still occurred, but it was very small (Table 2). Nested FHLUs (e.g., the terraces in Figures 6 and 7 and all wetland depressions) increased the number of upward breaks in slope and amplified this effect. Depressional wetlands added to the Riverine Valley and Coastal Terrain hydrologic landscapes, created flow divides, and caused all groundwater flow to discharge to the surface in the two-dimensional plane we simulated (Figures 6 and 7).

There exists an interesting question of what happens to the groundwater that rises toward the surface. At a basic level, the water rising to the surface either evaporates, is transpired, or becomes surface water and continues downgradient. Groundwater moving toward the surface may be intercepted by plants and transpired or may evaporate as it nears and reaches the surface. In many cases, ET lowers the water table and helps prevent groundwater discharge to the surface. In this case, groundwater may rise to the water table and flow laterally as groundwater until the water table intersects the surface. Finally, if the water table coincides with the surface, groundwater may discharge to the surface and become surface water. We believe the water table in the hydrologic landscape figures in Winter [23,27] reflects this process.

**Figure 8.**
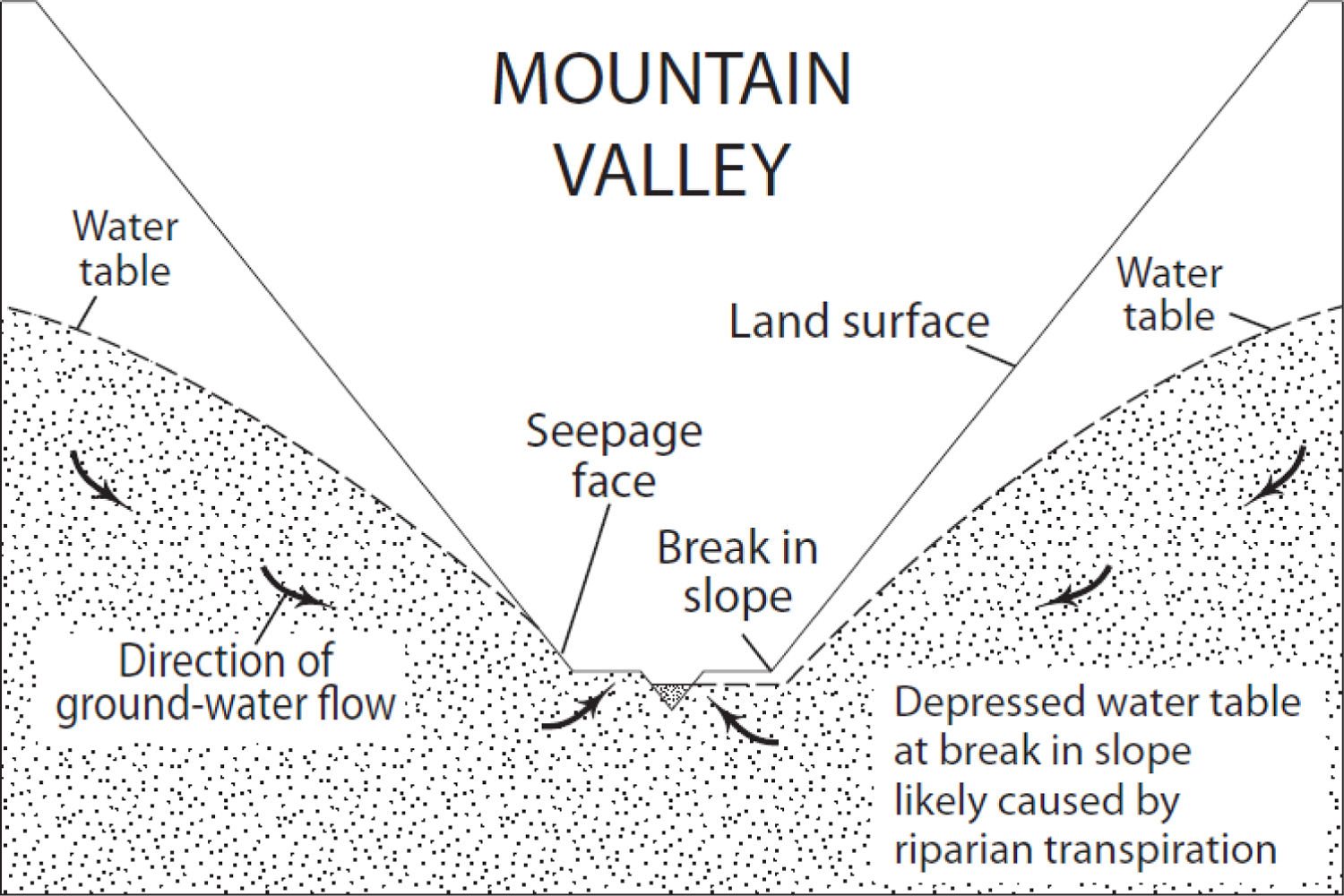
Mountain Valley hydrologic landscape modified from Winter [23].

For example, the mountain valley landscape figure from Winter [23] is reproduced here as Figure 8. Note that a seepage face is depicted for one side of the valley but not the other. This feature of the mountain valley landscape figure is not directly addressed in either Winter [27] or Winter [23]. However, since the landform is identical on either side of the valley, we may implicitly interpret the difference in water table as being caused by either geology or climate conditions. Given the apparently homogenous geology throughout the landscape, climate conditions are the most likely suspect; in this case, riparian transpiration provides a plausible explanation for maintaining the water table beneath the surface. The seepage face on the left side of Figure 8 likely indicates diminished ET that is less than the quantity of groundwater moving toward the surface. On the right side of Figure 8, the water table is maintained beneath the surface and groundwater flow is forced to continue downgradient where it discharges directly to the waterbody at the landscape lowland.

Our models accommodated ET indirectly, by using a recharge boundary in upland areas equal to net recharge, defined as precipitation less ET. We did not simulate spatial heterogeneity in ET. Commonly, especially dense vegetation exists at the fringes of wetlands and causes a depression in the water table at the edge of the wetland pool, actually drawing water out of the wetland pool toward the root zone on the periphery of the wetland [41] (see especially Figure 7). Since we did not simulate this phenomenon, the seepage faces in our simulations may better be interpreted as areas where groundwater discharge to the wetland may occur, but not necessarily to the pool of water within the wetland. Hayashi, et al. [17, see especially Figure 2] provided a useful explanation of the distinction between the spatial extent of a wetland and the extent of the pool of water within a wetland.

If groundwater upwelling near the upward break in slope manages to avoid being lost to ET and reaches the surface, it becomes surface water and continues downgradient. This surface water may also be lost to ET before flowing to a downgradient waterbody, or it may even return to the groundwater system as recharge. It is best to think of groundwater that discharges to the upward break in slope as *possibly* being connected to downgradient waterbodies. We interpret the groundwater that discharges to the surface in our models to represent the potential magnitude of connectivity through this route. From a management perspective, this is worth considering. Groundwater discharge to upward breaks in slope has the potential to transform groundwater connectivity to downgradient waterbodies from a direct groundwater connection to an indirect connection via surface-water. Callahan, et al. [42] described this exact process in action, showing that much of the nitrogen-rich groundwater from alder-covered hillslopes is first discharged to and modified by toeslope wetlands in salmon-bearing headwater streams in Alaska.

### 4.2. Other Support for Study Findings

The conceptual modeling approach taken in this study has distinct limitations. However, alternate lines of reasoning support the conclusion that a large portion of groundwater may discharge to upward breaks in slope rather than flow directly to downgradient waterbodies. First, a wealth of literature documents groundwater discharge to the surface at areas near upward breaks in slope, far away from downgradient waterbodies. This type of groundwater discharge is responsible for stream baseflow, which can be a large portion of total streamflow [43–45]. The literature on this point is so abundant that our contribution here is merely to draw attention to the importance of breaks in slope and the potential magnitude of this type of hydrologic connection.

**Figure 9.**
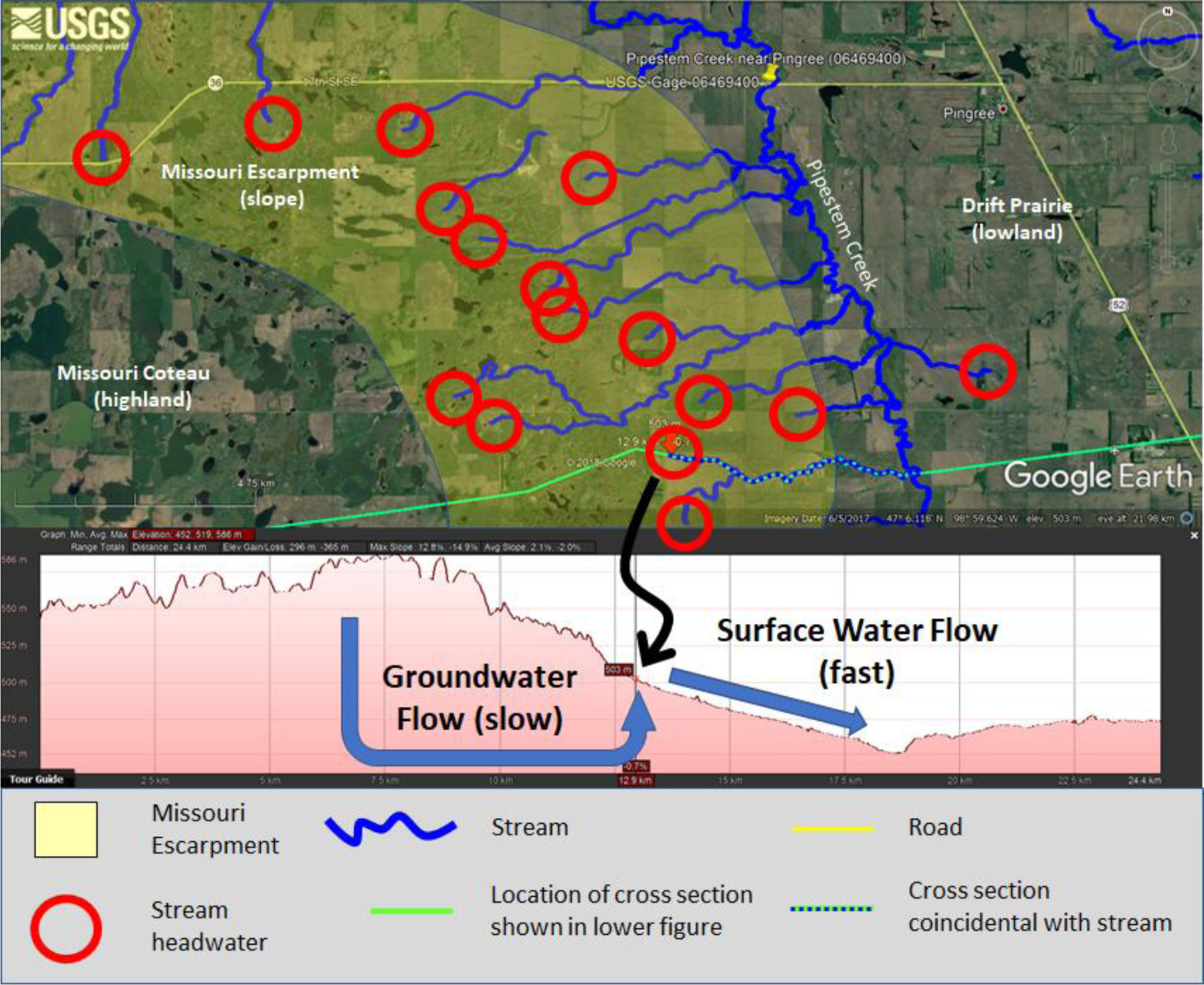
Annotated Google Earth screen capture illustrating the flow of water across a landscape near Pingree, ND. At bottom is an elevation profile of the cross section drawn through the image. The position of the headwaters of a tributary creek in the cross section is indicated on the cross section by the black arrow at a major upward break in slope.

A second line of reasoning to support our conclusions with regard to groundwater discharge at upward breaks in slope is provided by observations of in situ hydrologic conditions. For example, we can observe that stream headwaters often occur at upward breaks in slope. Figure 9 provides an example of stream headwaters occurring at an upward break in slope, likely indicating locations of groundwater discharge. In this example, water flows from the Missouri Coteau highlands, at the left of the figure, past the Missouri Escarpment, which slopes downward from west to east, to the Drift Prairie lowlands at the right. Groundwater recharge likely occurs primarily on the Missouri Coteau, and primarily discharges near the upward break in slope on the Missouri Escarpment. Water that discharges near the break in slope often contributes to the headwaters of streams that flow to Pipestem Creek as surface-water flow, which we describe as indirect groundwater connectivity to Pipestem Creek. This process reduces the length of the relatively slow groundwater flowpath and introduces a much faster surface water flowpath, which can be expected to greatly reduce the time required for groundwater recharge from the Missouri Coteau to reach Pipestem Creek.

Recently, Brooks, et al. [46] conducted a water isotope (δ^18^O and δ^2^H) study in this area that confirms the general assertions in the previous paragraph and refines the likely flowpaths in this particular setting. These authors found that the majority of water within Pipestem Creek originated from isotopically depleted groundwater. Isotopic variance within surface water throughout the watershed was related to the extent of evaporation of water residing in depressional wetlands, supporting the idea that direct and indirect pathways of groundwater dominated the flow in Pipestem Creek. The proportion of unevaporated water (groundwater with low residence time in the surface water system) increased in the downstream direction, indicating that wetland storage of groundwater was more important in the upper parts of the Pipestem watershed. The tributaries draining the Missouri Escarpment also showed the distinct groundwater signal, but they varied greatly in the degree of evaporation. However, they did not vary isotopically with precipitation inputs, indicating groundwater was the primary water source.

### 4.3. Consequences of Groundwater Discharge at the Upward Break in Slope

Groundwater discharging directly to tributary streams from adjacent uplands has profound consequences. This flow path maintains stream baseflow [45], moderates temperature [44,47], provides thermal refuge for aquatic species [48], and supplies nutrients to streams [42].

The magnitude of groundwater discharge at upward breaks in slope can enhance pond permanence of depressional wetlands and may support fill and spill or fill and merge surface water connectivity. For example, springbrooks are floodplain wetlands that receive groundwater discharge, which then flows overland to a nearby stream [49]. Much of the groundwater discharge to springbrooks may originate as regional groundwater flowing from nearby uplands, often flowing over long distances [50,51].

The effect of groundwater discharge on downstream waterbodies is affected by the physical flow path taken to the downstream waterbody, as well as chemical and biological processes along that flow path [52]. Groundwater discharge to the upward break in slope often supports wetlands, headwaters of streams, and stream baseflow [44]. Farther downstream, the chemical and physical makeup of this water is attenuated by many processes that are notable. For example, evaporation effects groundwater that discharges to the surface and continues downgradient. This alters the isotopic signature of the groundwater source and Brooks, et al. [46] used this evaporation signal to estimate the importance of surface water storage on downstream flows.

The groundwater-discharge-supported surface flows from breaks in slope to downgradient waters provide pathways along which aquatic biota can move, thereby influencing a landscape’s biotic connectivity via surface flows. However, while two-dimensional models like the ones we used in our simulations may be sufficient for elucidating the general aspects of water flows within a given FHLU, due to the fact that the movements of many organisms are not limited to aquatic pathways (e.g., adult amphibians, flying insects [53]), these models are overly simplistic in terms of identifying wetland connectivity that can result from the multidirectional movements of biota. However, such water flow models are needed to inform biotic connectivity models. Mushet, et al. [54] described what they called “freshwater ecosystem mosaics” to facilitate the marriage of models that consider movements of water, materials, and/or biota along specific water-flow pathways (e.g., streams, rivers), linking individual waterbodies (e.g., wetlands, lakes) with ecosystem models that include exchanges of energy, nutrients, materials and organisms between the aquatic features and surrounding upland areas. The knowledge gained from the use of two-dimensional FHLUs can be used to inform the transition to three-dimensional models that include the spatial distribution of water bodies, interconnecting flows, and the myriad influences of the upland areas between, i.e., all of the components of a complete freshwater ecosystem mosaic.

In addition to providing surface-water pathways along which biota can move and therefore support biotic connectivity, the substantial groundwater discharge that can occur at a break in slope can lead to unique communities occurring in these areas, thereby supporting biotic diversity [55]. For example, slope wetlands (e.g., fens) that do not pond water can occur at these slope breaks [2,56]. In places such as the prairie pothole region, slope wetlands and the plant and animal communities they support are much rarer than the depressional wetlands used in our simulations. The presence of slope wetlands created by the conversion of groundwater to surface flows at breaks in slope can add greatly to an area’s overall biotic diversity [57].

Cumulatively, the magnitude of groundwater discharge at upward breaks in slope provides a potentially powerful connection from recharge areas, possibly including depressional wetlands, to downgradient waters. This point is easily lost when considering the hydrologic connectivity of wetlands to downgradient waterbodies as either a surface water (fill and spill) or groundwater (direct discharge) process.

### 4.4. General Patterns of Wetland Connectivity and Pond Permanence

The results illustrate that the elevation of the ponded-water surface of a depressional wetland relative to the elevation of the surrounding water table is the crucial variable that determines how the wetland pond is connected to other aquatic features on the landscape via groundwater. Where the surface of a wetland is above the surrounding water table, the wetland recharges the aquifer and groundwater flows away from it. Conversely, where the ponded-water surface of a wetland is beneath the surrounding water table, the wetland will normally receive groundwater discharge from all sides. Where the water table intersects the ponded-water surface of a wetland, the wetland will normally act as a flow-through wetland, both receiving and recharging groundwater (Table 3). These findings are consistent with the other literature [18,58]. Temporal variability in the balance between precipitation and evapotranspiration also causes many wetlands to transition between supplying groundwater recharge, receiving groundwater discharge, and serving as flow-through wetlands [59]. No exceptions to this general pattern were observed in this study, but Neff and Rosenberry [16] provided a summary of special conditions that can lead to exceptional cases.

Whether a wetland receives or contributes to groundwater has profound implications for the nature of a depressional wetland’s connectivity to the landscape and pond permanence. Wetlands that recharge groundwater (e.g., those with a ponded water level above the surrounding water table) are connected by groundwater to other parts of the landscape in the downgradient direction only. These wetlands also continually lose water to the groundwater system, are more likely to have low pond permanence [60,61], and may be less likely to fill and spill or fill and merge [5].

Depressional wetlands that receive groundwater discharge (e.g., those with a ponded water level below the surrounding water table) are connected by groundwater to other parts of the landscape in the upgradient direction only. These wetlands continually gain water from the groundwater system, are more likely to have high pond permanence, and are more likely to fill and spill or fill and merge.

Depressional wetlands that both receive and recharge groundwater are connected by groundwater to other parts of the landscape in both the upgradient and downgradient directions. Groundwater may not significantly affect the pond permanence or fill and spill and fill and merge behavior of these wetlands.

In concept, groundwater discharge at upward breaks in slope may also provide a more subtle source of water to help tip the water balance of a depressional wetland toward filling and spilling or merging. However, the current analysis was not intended to evaluate fill and spill or fill and merge. In particular, there are two limitations that make it difficult to examine this issue rigorously. First, fill and spill and fill and merge can be associated with high precipitation conditions in combination with high antecedent moisture or wet periods such as the spring snowmelt [13,62,63], conditions that can last for years due to possible regime shifts [5]. Since the present analysis used long-term average annual conditions, fill and spill and fill and merge that occur from shorter-term high precipitation events or periods are not included. Second, the current analysis has a limited ability to differentiate between fill and spill vs. fill and merge, since the scenarios do not include pairs of nearby wetlands. However, note that in both cases the existing model could be run in such a way in the future as to address these issues—first, by including higher frequency precipitation conditions and, second, by including scenarios which include pairs of nearby wetlands and including upstream contributions. This would provide insight into which aspects of hydrologic landscapes have a greater groundwater influence on wetland fill and spill or fill and merge and the conditions leading to that occurrence.

### 4.5. Key Variables Affecting Connectivity

Our results provide insight to conditions and processes that are particularly impactful on the orientation of a given depressional wetland to the surrounding water table. The actual conditions on the landscape vary and are dependent on several key variables.

#### 4.5.1. Climate and Geology

In the hydrologic landscapes theoretical framework, the water-table elevation of a given landform is a product of climate and geology. With regard to climate, areas with high groundwater recharge and low evapotranspiration facilitate a high water table relative to areas with low groundwater recharge and high evapotranspiration. With regard to geology, less permeable materials slow the gradient-driven flow of groundwater and raise the water table relative to coarse, more permeable materials. In this study, we held climate and geology as constant as possible in order to focus on the effect of landform on hydrologic flow through diverse landscapes; we recognize this intentional limitation.

Short-term timing of weather-based infiltration may cause the water table to fluctuate around a given depressional wetland. This may cause individual depressional wetlands to flip from recharge to flow-through to discharge wetlands, or possibly the reverse. This will alter the connectivity of *individual wetlands* with the landscape. However, it does not alter how each *type* of wetland is hydrologically connected to the landscape. In other words, the connectivity patterns of discharge, recharge, and flow-through wetlands will not change, even though individual depressional wetlands may shift between these states due to short-term fluctuations of the water table.

#### 4.5.2. Depth of Wetland Basin

The depth of the depression in which a wetland sits affects hydrologic connectivity in two ways. First, the deeper the depression, the more likely it is that the bottom of the depression will be below the surrounding water table. When combined with ET from the wetland, this scenario causes groundwater to flow toward a wetland and isolates it from downgradient waterbodies with regard to groundwater flow. The constant discharge of groundwater to these wetlands enhances the permanence of their ponded water.

With regard to surface-water connectivity, deeper depressions are less likely to be connected to downgradient waterbodies by fill and spill, for two reasons. Deeper depressions are inherently larger in volume than shallow depressions of a similar width-to-height ratio and will require the addition of a greater volume of water before spilling occurs. This effectively reduces the likelihood that fill and spill will occur. It is notable that deeper depressions are more likely to extend below the surrounding water table, causing groundwater discharge to the wetland. This condition would likely increase the inflow to a given wetland. However, as the deep-depression wetland fills, the surface of the ponded water will likely eventually rise to the same elevation as the surrounding water table. Once this occurs, groundwater discharge to the wetland will cease. The combined effect of deeper depressions is to make a depressional wetland less likely to fill and spill.

### 4.6. Special Conditions that Affect Groundwater Connectivity

The water table is often considered to be a subdued replica of the surface. It is tempting to make this simplifying assumption when developing hypotheses of hydrologic connectivity, especially given the difficulty of measuring the water table directly. Unfortunately, the water table fluctuates independently of the surface under many conditions. It is important to recognize these conditions to avoid incorrect judgements of connectivity.

#### 4.6.1. Tertiary FHLUs and the Water Table

Our simulations revealed certain arrangements of landforms cause the water table to fall and not follow the surface contour. Nesting of a depressional wetland within a broader hydrologic landscape often creates this specific arrangement. Figure 1 depicts two types of tertiary FHLUs, captured in inset boxes and labeled as tertiary FHLUs, both of which cause this unusual water table situation in our simulations. Note that the slope in both tertiary FHLUs is a concave-down land surface that actually rises from the wetland in the FHLU highland to the top of the wetland depression and then falls to the FHLU lowland. We refer to this slope as a ‘rising then falling slope.’

If the water table beneath this type of tertiary FHLU falls far enough, the highland wetland will either recharge groundwater or become a flow-through wetland by receiving groundwater discharge on the upgradient side and recharging groundwater on the downgradient side. Examples of this type of flow-through connectivity include Wetland 2 in the Playa 2 and 3, Plateau 2 and 3, and Mountain Valley 2 landscapes (Figures 3–5).

Our simulations show the water table dropping beneath this type of tertiary FHLU is sometimes not sufficient to change the connectivity pattern of the wetlands. Examples of this case include our simulations in the Riverine Valley and Coastal Terrain landscapes. We believe the magnitude of water table decline is reduced in cases where (a) the width of the rising then falling slope is especially wide, (b) the climate is relatively wet, and (c) low-permeability geologic substrate is present. Rosenberry and Winter [59] provided a field study of hydrologic connectivity for two adjacent prairie pothole depressional wetlands separated by a rising then falling slope.

**Figure 10.**
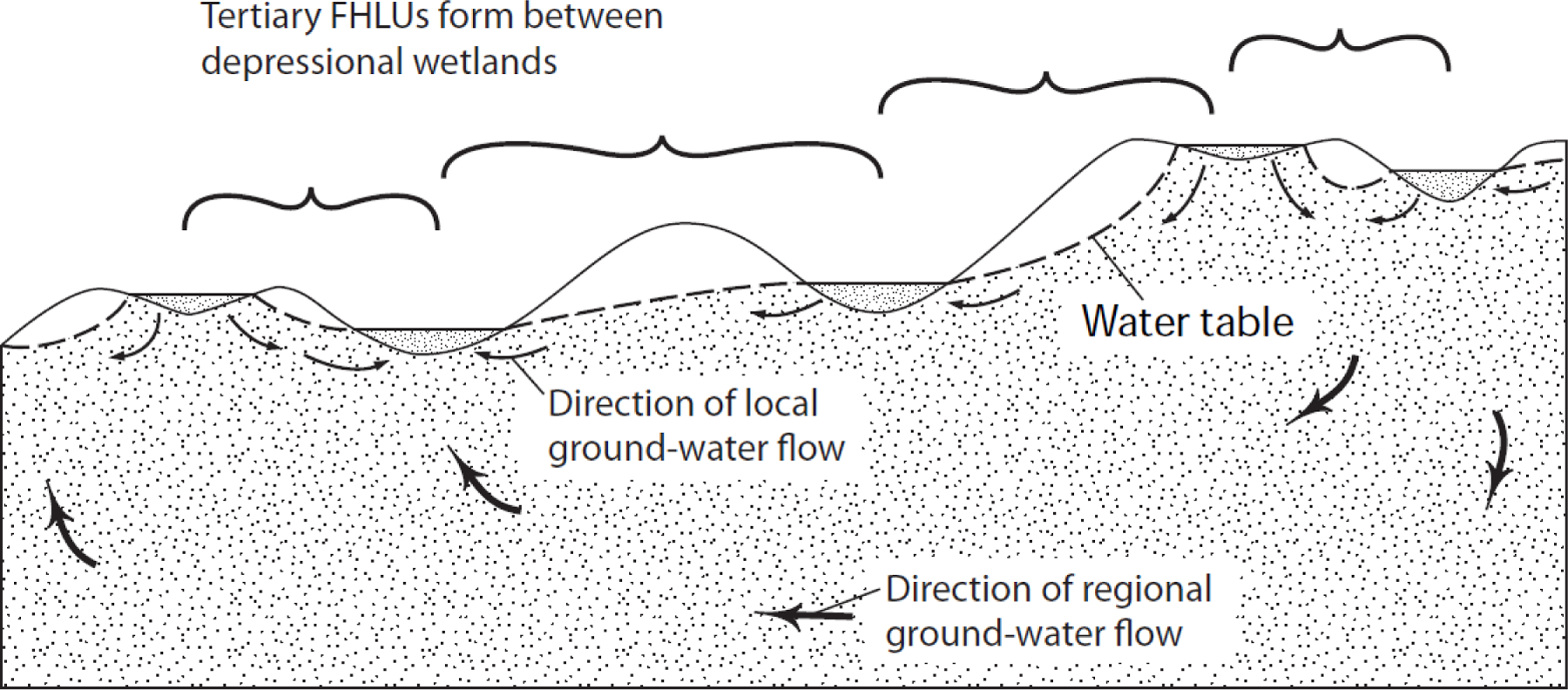
Groundwater flow through a hummocky terrain hydrologic landscape, tertiary FHLUs indicated by brackets. Modified from Winter [23,27].

Evaluation of the flow depicted in the hummocky terrain landscape from Winter [23,27] provides an additional example of the significance of a tertiary FHLU with a ‘rising then falling’ slope (Figure 10). In this landscape, the primary FHLU has a highland at the right edge of the landscape and a lowland at the left edge of the landscape. The slope dividing the highland and lowland is interrupted by five depressional wetland secondary FHLUs. Four tertiary FHLUs exist in this landscape, between the depressional wetlands, as shown in Figure 10. All of these tertiary FHLUs have a rising then falling slope, and the water table is depressed to varying degrees. In some cases, the water table falls sufficiently to allow groundwater to flow away from a depressional wetland. In one case, the second depressional wetland from the left, the depression serves only as a lowland in the tertiary FHLUs and therefore receives groundwater discharge. The hydrologic consequence of having many depressional wetlands embedded on this landscape is to cause the water table to not represent a subdued version of the surface contour, which creates complex flow paths and groundwater connectivity.

#### 4.6.2. Additional Situations Where the Water Table Contour Does Not Follow the Land Surface

The simulations done in this study reflect cases where the contour of the water table generally follows the land surface. However, there are situations where this does not hold true. Focused recharge, geologic heterogeneity, and riparian ET all can cause the water table to fall away from the land surface in specific locations [23]. Tile drains are often added to the landscape specifically to cause the water table to assume a shape that is the reverse from the surface contour and allow wet areas to become dry. Also, if the climate becomes especially dry, such as is the case with intense drought, reduced recharge and increased evapotranspiration could lower the water table sufficiently to prevent the water table from following surface contours. Likewise, a sufficiently porous substrate could allow a groundwater to drain and lower the water table sufficiently not to follow surface contours. Assuming the water table is a subdued replica of the surface in any of these cases can lead to the misinterpretation of groundwater flow and connectivity.

Hydrologically, these processes have two notable effects. First, breaks in slope on the surface may not reflect the contour of the water table below. This creates a case where groundwater flow must be judged by observing the water-table contours rather than using the landscape surface as a proxy for the water table. Second, these processes lower the water table and tend to dry out the ‘watery patches’ important for biodiversity (e.g., metapopulation migration moments, see [54]).

#### 4.6.3. Situations Where Groundwater Flow Does Not Follow Water Table Contours

Both geologic heterogeneity and strong anisotropy can cause groundwater to flow from a wetland, even if a water-table mound is present, to downgradient waterbodies [16]. These factors are particularly important when the water-table mound is especially small, the slope of the water table on the downgradient side of the mound is relatively steep, or the elevation drop to a discharge point is especially great [28,29,64].

### 4.7. Guidance for Practitioners

A key dilemma for practitioners is to sufficiently assess both direct and indirect wetland groundwater connectivity with limited resources. Golden, et al. [35] made the case that measured, modeled, and hypothesized information can be creatively combined depending on resource availability to provide a defensible basis for decision making. Each form of information has its advantages, complements the other two, and can be pursued to varying degrees.

**Figure 11.**
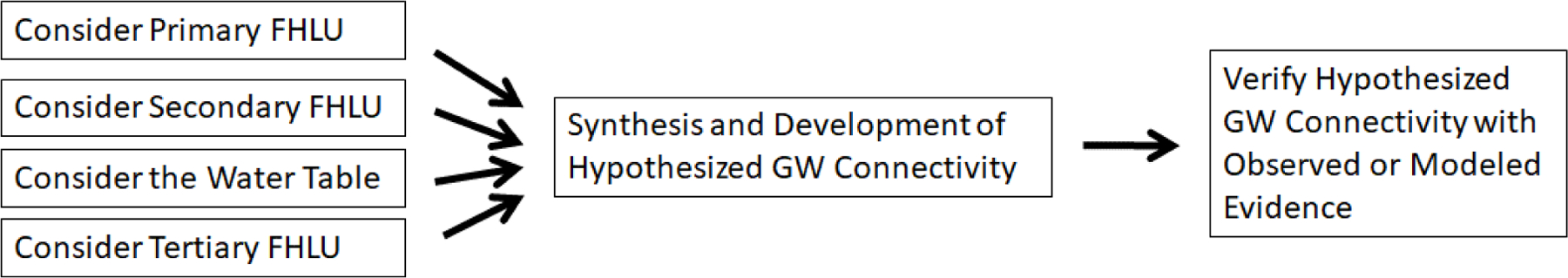
Flow chart to assess depressional wetland connectivity.

One contribution of this study is to improve the theory available to practitioners to develop a vision, or hypothesis, of groundwater connectivity on the landscape before employing measurements or modeling. This may constrain the information needed from measurements or modeling, or it may inform more cost-effective ways of employing those approaches. Figure 11 provides a simple workflow that uses hypothesized connectivity to efficiently build an understanding of wetland connectivity via groundwater.

A good first step when developing a hypothesized vision of the groundwater connectivity of a landscape is to assess the primary FHLU. To do this, first identify the primary landform, without regard to small-scale local relief. It is useful to visualize the ‘default’ water-table contour. The tendency is for the water table to roughly follow the land surface in a subdued manner, with some exceptions. First, downward breaks in slope cause the water table to decline locally. This creates an opportunity for wetlands in these upland settings to serve as focal points for groundwater recharge (e.g., [58]). Second, upward breaks in slope cause the water table to rise toward or to the surface, where the large majority of groundwater discharge and contribution to surface-water flow occurs. In many scenarios, the key groundwater connection in a landscape is the indirect groundwater connection rather than the direct groundwater connection. Finally, geology and climate will further modify the water table and flow through a hydrologic landscape and existing knowledge of these parameters can be used to define the primary FHLU (Figure 1).

Identifying embedded, secondary FHLUs is the second step to develop a hypothesis of wetland groundwater connectivity. These can be relatively major landscape features, such as terraces, or relatively small features such as depressional wetlands. With regard to depressional wetlands, the size and depth of a depression are important to consider as key factors that affect groundwater connectivity (Table 3).

The third step in developing a hypothesis of wetland groundwater connectivity is to consider the predominant behavior of the water table (Table 3). Shallow well data tracking the water table elevation are ideal, but usually not readily available. Fortunately, the water table can be evaluated as creatively as funds, time, and available expertise allow and need not be expensive. In particular, a simple visual examination of the landscape features may prove sufficient to develop a hypothesis of the predominant water table behavior. For example, the presence of stream headwaters or springs often indicate groundwater discharge (Figure 9). The presence of biota adapted to highly mineralized waters often indicates strong regional groundwater inflow [65,66]. Historical aerial and satellite imagery, such as imagery available freely on Google Earth, can provide insight to pond permanence and response to seasonal or longer-term climate variability. Place names can also be relevant, examples include features such as Dry Creek or Great Sulfur Spring. The lack of, or flooding of, residential basements may provide information on water table behavior. Peculiar streamflow changes between wet and dry years may indicate a high degree of fill and spill connectivity, as described in Shaw [14] and Shaw, et al. [67].

Assessing the geology of the area is vital to understand the predominant water table behavior. Our results were obtained by assuming geological homogeneity and isotropy, to isolate the independent effects of the landform, especially when depressional wetlands are introduced. However, geological heterogeneity and anisotropy are the norm, and have profound effects on water-table shape, groundwater flow direction, and associated hydrologic connectivity [68]. For example, vernal pool wetlands of similar size, shape, and spatial arrangement, but formed in different geological settings, can have vastly different physical and chemical hydrological characteristics, with some connected to the landscape only by surface-water fill and spill and others being connected both by groundwater flow-through and surface-water fill and spill [69]. Fortunately, highly detailed geologic maps and literature are available in many areas. Well logs, observations of bedrock outcroppings, or the presence of sand or gravel mining can also provide insight to key aspects of the surrounding geology.

Similarly, evaluating the climate and its variability is essential to understand the predominant water table behavior. Our study evaluated steady state conditions with homogenous and constant infiltration. Real settings are dominated by spatial and temporal variability in precipitation and evaporation, which drives variability of infiltration. These processes greatly affect the water table. To aid in this evaluation, weather station data are often readily available and can provide much information about precipitation amount, type, and seasonality, as well as the evaporation rates in an area.

The fourth step to develop a hypothesis for wetland groundwater connectivity is to identify tertiary FHLUs and how they may cause exceptions to the predominant behavior and location of the water table. The presence of one or more depressional wetland creates an especially important type of tertiary FHLU, characterized by a slope between upland and lowland that rises to the top of the wetland depression then falls to the lowland, as depicted in the two tertiary FHLUs in Figure 1. In our simulations, this type of FHLU is especially likely to have a water table that does not follow the land surface contour and caused depressional wetlands in areas normally associated with groundwater discharge to the surface to act as flow-through wetlands with a groundwater connection to downstream waterbodies. Example of this type of connectivity include Wetland 2 in the Plateau 2, Plateau 3, and Mountain Valley 2 landscapes (Figures 4 and 5).

A synthesis of the FHLUs, climate, geology, and readily observed evidence of the water table should provide a hypothesis for how depressional wetlands are hydrologically connected to the landscape. From this point, hydrologic modeling and/or the addition of new observations, such as from well-placed piezometers, can confirm or refine the hypothesis of groundwater connectivity of depressional wetlands.

The value of evaluating the hydrologic landscape to develop a connectivity hypothesis is to quickly and inexpensively simplify, improve, and reduce our dependence on hydrologic modeling or installing new monitoring equipment for decision making. For example, a relatively large modeling effort, or extensive installation of monitoring equipment such as piezometers and streamflow gauging stations, may provide good enough information to confidently make land-use management decisions. However, these approaches may be relatively expensive, uncertain, time consuming, or demand expertise that is not readily available. By developing a high-quality hypothesis of connectivity, the modeling and collection of new measurements or observations can be done selectively and merely to confirm the hypothesis. In addition, a convergence between hypothesized, modeled, and observed information improves confidence that we correctly understand groundwater connectivity in the study area.

The authors used a less refined version of the process depicted in Figure 11 when siting piezometers and water quality sampling locations in a prior study [46]. In this example, the study area was in the prairie pothole region of North Dakota and included the area shown in Figure 9. One aspect of the example study was to evaluate the potential for groundwater connectivity between depressional, ‘pothole’ wetlands on the Missouri Coteau highlands and Pipestem Creek on the Drift Prairie lowland. The study area was conceptualized as being similar to a plateau hydrologic landform and two particular locations were targeted for piezometer nest installations and water quality sampling sites; the upward break in slope on the Missouri Escarpment and the bank of Pipestem Creek. The exploration of the study area revealed stream headwaters and one spring at the upward break in slope on the Missouri Escarpment, tentatively confirming the presence of groundwater discharge in this area. Piezometer nests were installed at prominent upward breaks in slope and near the bank of Pipestem Creek and monitored. A groundwater modeling exercise used these data and confirmed these locations as likely sites for groundwater discharge. Water quality analyses of groundwater samples at these locations verified the nature of discharging groundwater as having originated from recharge in upland areas and not pothole wetlands. In this example, the use of a groundwater connectivity hypothesis guided field instrumentation and modeling and the combination of all three sources of information provided greater confidence in the study conclusions.

## 5. Conclusions

Simulated flow through five generic hydrologic landscapes quantitatively shows that most groundwater flow from uplands to lowlands is indirect, discharging first to the surface at an upward break in slope and then continuing downgradient as surface-water flow. In essence, upward breaks in slope transform the nature of groundwater-connectivity across a landscape from a direct groundwater connection to, predominantly, an indirect groundwater connection. In addition, embedding depressional wetlands within a broader landscape introduces additional upward breaks in slope that serve to amplify groundwater flow toward the surface and restrict regional groundwater flow. The presence of depressional wetlands within a broader landscape also introduces additional downward breaks in slope that serve to lower the water table locally. In some cases, particularly on the downgradient side of a depressional wetland, a landform occurs that rises to the top of the depression, then falls to the lowland. The water table in this area is especially prone to not following the contour of the land surface and instead slopes monotonically away from a depressional wetland, causing it to be groundwater-connected to downgradient waterbodies. With regard to water-resource management, we explain how applying a hydrologic-landscapes approach to developing a hypothesis of groundwater connectivity can be used to improve practitioners’ understanding of wetland connectivity and guide more resource-efficient measurement and modeling efforts to support decision making. Finally, this work provides a framework for using the hydrologic landscapes theory to understand wetland connectivity via groundwater.

In situ landscape complexity presents a key limitation of our analysis. In particular, our focus was on the role of landforms in determining groundwater connectivity and we intentionally restricted complexity related to climate and geology. Understanding all the nuances of landscape-scale connectivity will likely never be possible, but our analysis is an important step in that direction. One role of this research regarding the groundwater connectivity of wetlands is to provide a basis to develop tools and guidance needed by decision makers. Future research can help shrink the remaining gap.

## Figures and Tables

**Table 1.** Cell sizes, net recharge, and geologic type used in each domain. Vert. is vertical, Horiz. is horizontal, Grad. is the regional topographic gradient. Parameters for generic geologic type followed by “CP” were documented in Carsel and Parrish [32], all others were described in Lappala, et al. [33]. Parameters were developed to replicate the water table and groundwater flow depicted in Winter [23] as closely as possible.

Domain	Dimensions (m)			Grid Cell Size (m)		Net Recharge (m/d)	Geology ^1^
	Vert.	Horiz.	Grad.	Vert.	Horiz.		
Playa	1000	9500	0.11	10	63.3	2.00 × 10^−4^	silty clay CP
Plateau	300	20,000	0.02	3	200	3.50 × 10^−5^	silt-loam
Mountain Valley	300	7000	0.04	1.5	20	3.50 × 10^−5^	silt-loam
Riverine Valley	250	10,000	0.03	0.75	20	3.50 × 10^−5^	silt-loam
Coastal Terrain	200	11,500	0.02	1	20	3.50 × 10^−5^	silt-loam

1 van Genuchten Class

**Table 2.** Percentage of groundwater that discharges near the upward break(s) in slope of each landscape, within 500 m or as specified.

Domain and Break	Discharge	Domain and Break	Discharge
Playa 1	90.06%	Riverine Valley 2 – 1st break to wetland 2	100.00%
Playa 2 - break to wetland #3	97.97%	Riverine Valley 2 – 2nd break to wetland 3	100.00%
Playa 3	91.99%	Riverine Valley 2 – 3rd break	48.12%
Plateau 1 – 600 m uphill to 500 m downhill of break	89.87%	Coastal Terrain 1 – 1st break	>> 99.99%
Plateau 2 – 600 m uphill to 800 m downhill of break	90.15%	Coastal Terrain 1 – 2nd break	>> 99.99%
Plateau 3 – 200 m uphill to 800 m downhill of break	87.76%	Coastal Terrain 1 – 3rd break	99.88%
Mountain Valley 1	99.95%	Coastal Terrain 2 – 1st break to wetland 2	100.00%
Mountain Valley 2 - Break to wetland 2	98.27%	Coastal Terrain 2 – 2nd break to wetland 3	100.00%
		Coastal Terrain 2 – 3rd break	99.89%
Riverine Valley 1 – 1st break	99.98%		
Riverine Valley 1 – 2nd break	99.84%		
Riverine Valley 1 – 3rd break	77.54%		

**Table 3.** Summary of general patterns of wetland connectivity.

Wetland Surface Relative to Water Table (Dashed Line)	Connectivity in Upgradient Direction	Connectivity in Downgradient Direction	Likely to Spill?	Likely Pond Permanence	Likely Location(s)
Above 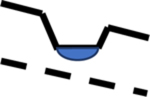	Surface water only	Groundwater; Surface water, if fill and spill occurs	No, unless depression is especially shallow	Low	Groundwater recharge areas, especially near a downward break in slope.
Below 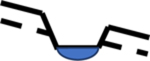	Groundwater and surface water	Surface water, if fill and spill occurs	Yes, unless depression is especially deep	High	Groundwater discharge areas, especially if not near downward breaks in slope.
Same 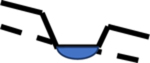	Groundwater and surface water	Groundwater; Surface water, if fill and spill occurs	Possibly	Moderate	Slopes, also groundwater discharge areas if near a downward break in slope.
